# Similarities and differences regarding acute anorexia nervosa and semi-starvation: does behavioral thermoregulation play a central role?

**DOI:** 10.3389/fnbeh.2023.1243572

**Published:** 2023-10-25

**Authors:** Lucille Lakier Smith

**Affiliations:** Human Performance Laboratory, Department of Kinesiology, School of Health Sciences, East Carolina University, Greenville, NC, United States

**Keywords:** hyperactivity (HyAc), shallow torpor, low body temperature (Tcore-low), cold sensations, foraging, evolution

## Abstract

**Objective:**

To clarify the association between acute anorexia nervosa (AN) and semi-starvation (SS) by focusing on similarities and differences in physiology, mood, and behavior.

**Method:**

A comparison of published literature between these two groups.

**Results:**

Both groups show similar hormonal and metabolic changes in response to caloric restriction and extreme weight loss (~25%). Associated changes result in a reduced body temperature (T_core-low_). Maintenance of body temperature within a specific range is crucial to survival. However, both groups cannot activate autonomic strategies to maintain their T_core-low_, such as increasing metabolic rate, constricting skin blood vessels, or shivering. Furthermore, T_core-low_ increases the individuals’ “coldness sensations” throughout the body, hence the frequent reports from ANs and SSs of “feeling cold.” To eliminate these uncomfortable “coldness sensations” and, importantly, to maintain T_core-low_, ANs, and SSs *“select” different thermoregulatory behavioral strategies*. It is proposed that the *primary differences* between AN and SS, based on genetics, now manifest due to the “selection” of different thermo-regulatory behaviors. AN patients (ANs) “select” hyperactive behavior (HyAc), which increases internal metabolic heat and thus assists with maintaining T_core-low_; in harmony with hyperactive behavior is a lively mood. Also related to this elevated arousal pattern, ANs experience disrupted sleep. In contrast, SS individuals “select” a passive thermo-behavioral strategy, “shallow torpor,” which includes reduced activity, resulting in energy conservation. In addition, this inactivity aids in the retention of generated metabolic heat. Corresponding to this lethargic behavior, SS individuals display a listless mood and increased sleep.

**Conclusion:**

Initial *similarities* between the two are attributable to physiological changes related to extreme weight loss. *Differences* are most likely attributable to genetically programmed “selection” of alternate thermoregulatory strategies, primarily to maintain T_core-low_. However, if acute AN is prolonged and evolves into a chronic condition, AN will more closely align with starvation and more precisely reflect SS symptomology.

## 1. Introduction

Anorexia Nervosa (AN) is a condition wherein an individual, usually 1–4% of females (Watson et al., 2019), self-imposes severe food restrictions and loses up to 25% of body weight. Over the decades, AN has been regarded principally as a *psychogenic condition*, but one with severe physiological consequences (Pirke, 1987; Dalle Grave et al., 2008; Treasure et al., 2015; Calugi et al., 2018; Watson et al., 2019). A related condition is semi-starvation (SS); this, too, manifests due to a striking reduction in food consumption and dramatic weight loss (Keys et al., 1950; Fichter et al., 1986). However, SS is typically in response to uncontrolled circumstances, such as famine, and is usually not willfully self-imposed. Furthermore, unlike AN, SS is regarded predominantly as a *physiological condition with associated psychological and behavioral changes* (Keys et al., 1950).

Many researchers have questioned whether AN represents a unique condition or whether AN reflects the sequelae associated with semi-starvation (Mecklenburg et al., 1974; Casper and Davis, 1977; Calugi et al., 2018; Phillipou et al., 2018; Frank et al., 2019; Casper et al., 2020a). Casper and Davis (1977) suggested that “the effects of starvation are more intimately involved in the symptoms of anorexia nervosa than has heretofore been described.” According to Garner (Garner, 1997), “One of the most important advancements in the understanding of eating disorders is the recognition that severe and prolonged dietary restriction can lead to serious physical and psychological complications. Many of the symptoms once thought to be primary features of anorexia nervosa are actually symptoms of starvation.”

To better understand and thus improve the treatment of AN, Phillipou et al., (2018) have suggested that it is crucial to determine which signs and symptoms are explicitly related to AN and which represent the condition of semi-starvation. The present paper will attempt to unravel these conditions by assessing the similarities and differences between AN and SS (Casper and Davis, 1977) and determining whether *acute* AN represents a unique condition with distinctive psychological and physiological parameters, whether it reflects semi-starvation and its sequelae, or is a combination of these two conditions (Tyszkiewicz-Nwafor et al., 2020).

The investigation of AN focuses primarily on published literature on *acute-restrictive AN* (Casper and Davis, 1977; Carrera et al., 2012), implying that the individual is close to the time of onset of significant weight loss (Casper, 2018). However, it is difficult to verify when exactly the AN condition manifested since, typically, many months or even years pass between the earliest changes in eating, the dramatic weight loss, and the formal diagnosis (Kay, 1953; Scolnick and Mostofsky, 2014; Frostad and Bentz, 2022).

Regarding SSs, the emphasis will be on the classic Minnesota Study of Semi-Starvation (MinnSS) conducted in 1943, during World War II (WWII). Keys and colleagues (Keys et al., 1950) selected 36 average-weight, healthy, intelligent, psychologically well-adjusted, young white males who could get along reasonably well under trying conditions. The study consisted of:

- a 3-month control period during which subjects maintained average body weight,
- a 6-month semi-starvation period (~50% of previous caloric intake), and
- a 3-month weight recovery period.

This paper will focus on the 6-month semi-starvation period, during which subjects lost ~25% of body weight, similar to losses seen in AN. A team of medical doctors monitored these subjects to understand behavioral, physiological, psychological, and social changes. In addition to the dramatic reduction in food intake, subjects were *required* to participate in the same scheduled activities *for the entire year*; the purpose was to simulate wartime conditions.

Many notable differences exist between the MinnSS study and AN (Keys et al., 1950). However, this remains the most comprehensive “control group” available. It provides impressive information and could not be conducted nowadays for ethical reasons.

## 2. The initiation of the starvation response in ANs and SSs: leptin

Leptin is a hormone synthesized and released from fat tissue with its main action occurring in the CNS/hypothalamus (Jequier, 2002; Muller et al., 2009; Hebebrand et al., 2022). Blood levels are usually proportional to the amount of body fat. In this manner, it acts as a messenger molecule to inform the brain of the status of *fat energy reserves* in the body (Jequier, 2002; Muller et al., 2009).

At the onset of caloric restriction and weight loss, blood leptin levels *decrease* rapidly and dramatically (Seoane-Collazo et al., 2020); these decreased levels play a major role in adapting the organism to starvation (Ahima et al., 1996; Hebebrand et al., 2023). Reduced leptin levels signal the hypothalamus of imminent “danger” related to reduced food availability (Ahima et al., 1996; Muller et al., 2009; Hebebrand et al., 2022). The hypothalamus-pituitary-target organ axes, in addition to other changes (Hebebrand et al., 2019), now increase or decrease the synthesis and secretion of many energy-intensive hormones (Misra and Klibanski, 2014; Schorr and Miller, 2017):

- It decreases the synthesis of reproductive hormones, thus suppressing reproductive-related activities (Casper and Davis, 1977; Misra and Klibanski, 2014).
- It decreases the release of thyroid-stimulating hormone and the activity of the sympathetic nervous system, which, among other functions, reduces metabolic rate (Fichter et al., 1986; Misra and Klibanski, 2014; Schorr and Miller, 2017).
- It reduces the synthesis of insulin-like growth factor, which then inhibits growth and development (Fichter et al., 1986; Schorr and Miller, 2017) in the face of food scarcity and thus assists with extending survival (Devlin, 2011).

These reduced energy needs for growth and reproduction diminish the organisms’ need to draw on the primary energy source, *stored fat reserves.*

Regarding ANs, leptin levels are consistently lower, by as much as ~75% compared to HC (ECKERT et al., 1998; HAAS et al., 2005; Dostalova et al., 2007; Karageorgiou et al., 2020). Also, depending on the degree and severity of the caloric deficit, leptin levels may be undetectable (Hebebrand et al., 1997).

Hebebrand et al. (2022) suggest that off-label treatment with recombinant human leptin (metreleptin) may alleviate many of the emotional, cognitive, and associated behavioral symptoms reflective of AN. They also propose that this treatment could possibly break the vicious cycle that sustains this condition (Hebebrand et al., 2023). Milos et al. (2020) treated three *severely* anorexic patients; this short-term treatment proved partially successful.

However, the use of this treatment remains controversial. Casper (2022) acknowledges that short-term high doses of metreleptin mitigated feelings of depression, inner tension, intrusive thoughts of food, and the urge to be physically active. Yet when treatment was terminated, this had little influence on the patients’ personal commitment to remain at a low weight. Fraga et al. (2020), using rats, argued that ambient temperature was more effective in reducing the amount of running and weight loss than metreleptin. Placebo-controlled clinical trials are needed to confirm or refute the usefulness of metreleptin in treating AN (Hebebrand et al., 2019).

Regarding SS: there is a *similar significant decrease in blood leptin levels* in obese subjects, and normal-weight males and females *due to famine, fasting, or willful caloric restriction.* This decrease varies from 44 to 83% (Boden et al., 1996; Considine et al., 1996; Chan et al., 2003; Muller M.J. et al., 2015).

In summary, weight loss in *ANs and SSs rapidly decreases blood leptin levels*, altering the hormonal profile and preparing the body to withstand starvation. Specifically, there is a decrease in hormones that typically promote increased energy utilization, such as growth and reproduction. It has also been proposed that administering a leptin analog may induce psychological and behavioral changes to assist in recovery; however, this awaits clarification.

## 3. The maintenance of body temperature in HCs, ANs, and SSs

### 3.1. Healthy controls

Most Healthy controls (HCs) maintain a body core temperature (T_core_) of 37°C regardless of race, gender, and geographic location. Sustaining this level within a narrow range is critical to survival as it optimizes bodily functions and prevents cellular damage (Rising et al., 1992). Sustaining T_core_ involves the use of internal metabolic heat, which is a by-product of all energy-producing processes (Landsberg, 2012). This heat is then retained in the body by constricting surface/skin blood vessels and insulating body fat. If sufficient metabolic heat is not produced and retained, “emergency” strategies may become activated. These include shivering, activation of specialized heat-producing brown adipose tissue, and piloerection (goosebumps). However, these emergency strategies are energy demanding. Thus, HCs will initially preferably engage in a more straightforward strategy, *thermoregulatory behavior*. Such behaviors include wearing a sweater or turning up a thermostat, to name a few. *In endotherms, thermo-behavior is the most effective and frequently utilized means of maintaining T_core_* (Romanovsky, 2007; Flouris, 2011)*. Thermo–behavior is our first line of thermal defense to prevent changes in core temperature* (Schlader, 2014).

### 3.2. An and T_core-low_

Unlike HCs, numerous studies have demonstrated that ANs have a significantly lower T_core_. These low values range from 35.4°C (Mecklenburg et al., 1974; Rising et al., 1992) to 36.6°C (Davies et al., 1979); this will be referred to as T_core-low_ instead of hypothermia since hypothermia implies a T_core_ below 35°C. It is also suggested that this T_core-low_ represents a *new set point* for the ANs; it does not threaten survival but is crucial for them to maintain this level to prevent further reductions, which may become life-threatening (Smith, 2021).

The primary cause of a reduced T_core-low_ is a *significantly lower metabolic rate, which is a key provider of body heat and thus helps maintain internal body temperature.* The basal metabolic rate for ANs is reduced by approximately 21% (Polito et al., 2000; Onur et al., 2005) to 32% (Bossu et al., 2007) compared to HCs; thus, they cannot produce sufficient heat to maintain the normal 37°C.

Furthermore, Oishi et al. (2013) using mice, have demonstrated that fasting and the induction of ketosis, in the presence of hypothermia, may further impact body temperature. Since ANs are in metabolic ketosis (McCue, 2010) in the presence of T_core-low_, it is possible that this further exacerbates a reduction in body temperature.

In addition to the reduced ability to produce body heat, ANs cannot effectively *retain* this heat due to their inability to constrict surface blood vessels and due to having less insulating fat (Chudecka and Lubkowska, 2016). Furthermore, they cannot activate secondary backup thermoregulatory responses such as shivering, piloerection, or activation of brown fat (Vigersky et al., 1977; Nishita et al., 1986; Bredella et al., 2012).

However, like most humans, *they can initiate the crucial tactic of thermoregulatory behavior* ; many different thermo-behavioral strategies are available. It has been proposed that the *primary thermo-behavioral strategy employed by ANs* is the generation of internal metabolic heat through *muscle movement* (Gutierrez and Vazquez, 2001; Smith, 2021). This behavior is most likely a result of genetic programming; AN is now considered a psycho-metabolic condition, with as much as 76% of this condition being genetically determined (Bulik et al., 2019).

In support of the hypothesis that ANs generate heat through movement, is extensive evidence demonstrating that most ANs engage in excessive amounts of physical activity (Beumont et al., 1994; Davis et al., 1994, 1997; Alberti et al., 2013; El Ghoch et al., 2013; Gummer et al., 2015; Casper, 2018). The generated metabolic heat (Vargas et al., 2019) assists with maintaining or increasing T_core-low_; it also aids the perfusion of warm blood to the extremities, especially the hands (Vigersky et al., 1976), a primary area for the detection of cold sensations (Smith, 2021).

Although many have claimed that the main reason for excessive movement in AN is to enhance weight loss (Kiezebrink et al., 2009), several rodent studies have suggested otherwise. They have demonstrated that in a rat model referred to as activity-based anorexia, excessive running, when paired with food restriction and weight loss, represents a drive to generate body heat to assist with maintaining T_core-low_ (Hillebrand et al., 2005; Gutierrez et al., 2006, 2008). It is proposed that the ANs’ excessive activity represents a similar function (Gutierrez and Vazquez, 2001; Smith, 2021). This thermo-behavioral strategy will be referred to as *hyperactivity* (HyAc); a more detailed discussion will follow (Section 5.1).

### 3.3. SSs and T_core-low_

Like ANs, SSs have a reduced T_core_ and metabolic rate (Keys et al., 1950; Taylor and Keys, 1950; Tucker, 2006); furthermore, they too are in a state of metabolic ketosis (McCue, 2010); if hypothermic, this too could lower body temperature (Oishi et al., 2013). It is assumed that they cannot generate the backup thermal responses of shivering, piloerection, and activation of brown adipose tissue, although this has not been investigated. But, like ANs and other humans, they can *“select” specific thermoregulatory behaviors*. It is proposed that their central strategy is the converse of ANs. SSs “select” a more passive strategy that focuses on *retaining* whatever internal heat has been generated and *passively gaining* external environmental heat. This is verified by Keys et al. (1950) focusing on the behavior of their semi-starved subjects and using anecdotal observations from famine and prisoner-of-war camps. This behavioral pattern will be called shallow torpor (See Section 6.1).

In summary, it is hypothesized that the *primary difference* between ANs and SSs is their “selection” of *different* thermo-behavioral strategies (Barakat et al., 2023) most likely genetically determined. So, while ANs engage in HyAc to generate internal metabolic heat, SSs “select” a more inactive strategy that focuses on *retaining* whatever internal heat has been generated and *passively gaining* external environmental heat.

## 4. “Feeling cold” *initiates* engagement in thermoregulatory behavior

### 4.1. ANs and SSs “feel cold”

“Feeling cold” has been a prominent complaint for ANs (Swenne and Engstrom, 2005; Calugi et al., 2018) and SSs (Keys et al., 1950). *Surprisingly, this does not appear to be consciously related to changes in the body T_core-low_* (Jacquot et al., 2014; Vargas et al., 2018). Instead, feelings of body coldness are derived from sensations in the peripheral skin (Romanovsky, 2014). These “unpleasant” perceptions are then transmitted to the brain/hypothalamus, motivating engagement in thermo-behavior to lessen this discomfort (Flouris, 2011; Jacquot et al., 2014; Vargas et al., 2018).

### 4.2. ANs and cold sensations

Many researchers have reported that cold intolerance is a common complaint among ANs and is usually unrelated to ambient temperature (Lampert and Lau, 1976; Luck and Wakeling, 1980; Schwabe et al., 1981; Silverman, 1983; Crisp, 1984a; Bhanji and Mattingly, 1991; Kurklinsky et al., 2011; Carrera et al., 2012; Das and Maiti, 2013). In addition to these overall feelings of coldness, ANs complain especially of having cold hands (Nishita et al., 1986). “Fingers, toes, hands, and feet were usually blue and cold” (Lampert and Lau, 1976).

Recently it has been demonstrated that the hand skin temperature of ANs is the *only* skin surface area significantly lower (~2°C) than that of HCs. Surprisingly, all other surface areas of the body are the same or warmer than that of HCs, implying that metabolic *heat cannot be effectively retained* (Chudecka and Lubkowska, 2016). Thus, the hands are most likely the primary source for generating overall body thermal sensations (Vargas et al., 2018; Smith, 2021), with the skin temperature of the wrist being the best predictor of thermal sensations and of *motivation* to initiate thermoregulatory behavior (Jacquot et al., 2014).

### 4.3. SSs and cold sensations

Like ANs, SSs suffer from extreme sensations of coldness. Keys et al. (1950) reported that cold temperatures were poorly tolerated during the starvation phase of the MinSS, despite the room’s adequate heating. “Many subjects slept under heavy blankets and wore extra clothing during the day in hot summer weather.” In addition, subjects always requested that their food, coffee, and tea be served unusually hot. To feel warm, “all the men had learned to have long, hot showers “(Franklin et al., 1948), and when possible, would bask in the sun (Tucker, 2006), and gather about the steam radiators (Keys et al., 1950).

In another food-restricted study, the Carnegie study (Keys et al., 1950), the researchers commented on the subjects’ extreme sensitivity to cold. They noted that these men’s hand temperature, like ANs, was approximately 2°C lower than that of HCs.

So, both ANs and SSs experience profound sensations of coldness, especially in the extremities, which act as “bait” to induce thermo-behavior and thus reduce this discomfort. Simultaneously, and most importantly, this thermo-behavior assists with maintaining T_core-low_. It will now be proposed that the *“selection” of thermo-behavior differentiates ANs from SSs.* However, before discussing these *differences,* it should be noted that both groups display *additional behaviors that are similar*, especially those related to hunger and eating (Table 1).

**Table 1:** Additional similarities related to hunger, eating behavior, self-mutilation, and libido.

**Similarities**	**Anorexic patients**	**Semi-starved individuals**
**Hunger sensations (Related to Leptin and Ghrelin Levels)**	**Experience unrelenting hunger sensations** (Garfinkel, 1974; Speakman and Mitchell, 2011 Schorr and Miller, 2017)	**Experience unrelenting hunger sensations** (Tucker, 2006; Olson et al., 2020)
**Leptin: the “satiety” hormone**	**A dramatic decrease in blood leptin with weight loss** (Eckert et al., 1998; Hebebrand et al., 2022)	**A dramatic decrease in blood leptin with weight loss** (Muller T.D. et al., 2009)
**Ghrelin: the “hunger” hormone**	**Significantly elevated** (Sodersten et al., 2016a)	**Significantly elevated** (Muller T.D. et al., 2015; Nuttall et al., 2016)
**Eating Behavior**	**Slow eating, cutting food into small pieces, toying with food, etc.** (Beumont et al., 1997; Garner, 1997)	**Slow eating, cutting food into small pieces, toying with food, etc.** (Keys et al., 1950; Garner, 1997; Tucker, 2006; Hebebrand et al., 2022)
**Obsessional interest in food**	**Immersed in food-related behaviors: thinking, reading, talking** (Vigersky et al., 1976; Garner et al., 1982; Beumont et al., 1997)	**Immersed in food-related behaviors: thinking, reading, talking** (Beumont et al., 1997; Tucker, 2006)
**Make strange concoctions, possibly a form of pica**	**Possibly related to nutritional deficiencies** (Casper et al., 1980; Beumont et al., 1997; McCue, 2010; Miao et al., 2015)	**Possibly related to nutritional deficiencies** (Franklin et al., 1948; Keys et al., 1950; Tucker, 2006; McCue, 2010)
**Hoarding/stealing food**	**Possibly a survival strategy** (Crisp et al., 1980; Yao et al., 2017)	**Possibly a survival strategy** (Keys et al., 1950; Tucker, 2006)
**Body image**	**Distorted body image** (Ciwoniuk et al., 2022)	**Distorted body image** (Tucker, 2006)
**Self-injurious/mutilation behavior**	**Some engage in self-mutilation** (Crisp, 1984b Yaryura-Tobias et al., 1995)	**Some engage in self-mutilation** (Tucker, 2006)
**Libido**	**A dramatic decrease in sexual-related interests/activities** (Crisp, 1967; Price et al., 2020)	**A dramatic decrease in sexual-related interests/activities** (Keys et al., 1950; Hebebrand et al., 2022)

## 5. ANs and “selection” of thermo-behavior which coordinates with mood and sleep

### 5.1. ANs and hyperactivity

It is well established that as many as 78% (Davis et al., 1994) of ANs engage in *excessive* amounts of physical activity during the acute phase of the disorder (Beumont et al., 1994; Davis et al., 1994, 1997; Alberti et al., 2013; El Ghoch et al., 2013; Gummer et al., 2015; Casper, 2018). This surprising display of stamina continues even after significant weight loss (Vigersky et al., 1976; Casper et al., 2020). Many researchers have claimed that the primary reason for this HyAc is due to the relentless pursuit of weight loss (Kiezebrink et al., 2009); however, this has been disputed (Gutierrez and Birmingham, 2020).

To illustrate this excessive engagement in physical activity, Davis et al. (1994) administered questionnaires to 45 ANs and reported the following: 78% described their physical activity during the *acute phase* of their disorder as “excessive;” 93% of the patients described their exercise as “out of control” and “compulsive;” 75% reported that during the period of maximum weight loss, physical activity increased as their weight and food intake decreased. There were frequent comments from the ANs, such as “All I was doing was exercising” and “The lower my weight got, the more energy I had.” Furthermore, this increased urge to move continued despite increased levels of physical fatigue (Casper et al., 2020).

Many terms have been used to describe this excessive activity (Davis et al., 1999; Meyer et al., 2008, 2011). However, the terminology used here, hyperactivity (HyAc), will reflect the *total daily activity* of ANs (Smith, 2021) and includes three overlapping categories:

- Excessive Exercise (ExEx), involves “formal” exercise such as walking/jogging, weight training, volleyball, yoga, etc.
- Non-Exercise Activity Thermogenesis (NEAT) includes *all* movements during awake periods (Levine, 2007), such as performing daily chores, attending school/work, etc.
- Spontaneous Physical Activity (SPA) involves fidgety, restless behavior (Belak et al., 2017).

ANs find many ways of producing daily movement *in excess*. The execution of all these movements produces body heat to varying degrees. The generated metabolic heat now assists with T_core-low_ (Gutierrez and Vazquez, 2001; Smith, 2021).

In addition to HyAc to maintain T_core-low_, ANs *use other means to maintain/increase body temperature*. This may include wearing extra clothing, sitting close to heaters, and drinking warm fluids (Strumia, 2013).

Furthermore, HyAc may produce added benefits (Garland et al., 2011; Giel et al., 2013). These include reduced appetite, decreased anxiety, prevention or reduction of depressive-like behavior, and increased feelings of well-being (Scheurink et al., 2010; Hicks et al., 2019).

Although it has been stressed that HyAc is primarily a strategy to maintain T_core-low_ (Gutierrez and Vazquez, 2001; Smith, 2021) many view HyAc in ANs as an *ancient behavioral pattern aimed at encouraging foraging or migration during times of food shortages* (Casper et al., 1991; Guisinger, 2003; Scolnick and Mostofsky, 2014). However, engaging in HyAc for foraging and maintaining T_core-low_ are *not* mutually exclusive. If the ancient AN felt compelled to move, this would have maintained T_core-low_ and enhanced her chances of finding food.

Using a well-established rodent model, the activity-based anorexia model (ABA), it has been demonstrated that providing heat to the “anorexic” ABA-rodent reduces activity and weight loss (Routtenberg and Kuznesof, 1967; Epling and Pierce, 1996; Hillebrand et al., 2005; Gutierrez et al., 2006; Vazquez et al., 2006; Roura et al., 2020).

Applying translational reasoning, it has been hypothesized that providing heat to ANs, *may reduce the compulsion to move* and thus assist with weight gain (Gutierrez and Vazquez, 2001; Smith, 2021). However, the effectiveness of heat application in human ANs, remains equivocal.

Gull (1997) was the first to report that warming the spine, wearing warm clothing, and being kept in a warm bed for a time would promote recovery in ANs. In 2001 Gutierrez and Vasquez (Gutierrez and Vazquez, 2001) successfully treated three *hyperactive* ANs on an outpatient basis using three different heat treatments. All three patients showed an immediate reduction in overactivity. When assessed 30 months later, BMI for all three had improved dramatically, and activity patterns remained reduced. However, these were case reports with no control group. Additionally, compliance could not be assessed because the study was conducted on an outpatient basis.

In a Randomized Control Trial (RCT), Bergh et al. (2002) assigned 13 ANs to a Treatment group. The treatment consisted of using a computerized device (a Mandometer) during lunchtime to monitor food intake, rate of eating, and satiety. In addition, they supplied external heat by having subjects rest in a warm room (up to 40°C) for 1 h after lunch and confined subjects to a wheelchair or allowed them to walk slowly in the clinic. The Control group (n = 16) received no treatment. The outcome was significantly different between the treated and control groups. Fourteen patients in the treatment group went into remission after a median of 14.4 months, and 75% remained in remission at 12 months. They concluded that their treatment had a beneficial effect but also suggested that it was unclear what contribution each modality made (Mandometer, heat, reduced movement?). In a subsequent study, Zandian et al. (2017) reported that applying heat (room at 32°C) for 30 min after lunch significantly reduced anxiety.

Birmingham et al. (2004) tested the hypothesis that heat would increase the rate of weight gain. They randomized 21 subjects to a Heat or Control Group and applied heat for ~4 h daily for 21 days using a heated vest. Subjects reported enjoying the heating experience but did not show an improvement in eating behavior. Surprisingly, exercise behavior was not monitored, so whether heat impacted physical activity is unknown. In addition, the average BMI of these subjects was 17.5, suggesting a borderline condition. Also, these subjects would be considered chronic ANs since AN had lasted for approximately 13.6 years. These and other design factors may have confounded their results.

Cerrato et al. (2012) hypothesized that *ambient/seasonal t*emperature would impact the amount of *hyperactivity* in acute AN; they monitored daily movement using an accelerometer for 3 consecutive days. They assigned 15 ANs to a Warm Group between Spring and Summer (April – October), during which the mean ambient temperature was 16°C. Activity levels of the Cold group were monitored between Autumn and Winter (October–April) with a mean ambient temperature of 4.5°C. The results demonstrated that the Cold Group was significantly more physically active than the Warm Group (*p* = 0.003). They concluded that keeping patients warm may be a beneficial treatment option for reducing hyperactivity, which is typically refractory to treatment.

The following research design is proposed to address the question of whether applying heat 24/7 for an extended period (possibly 3 weeks–3 months) would assist with rehabilitation by *increasing the ANs T*_*core-low*_, *reducing* aspects of *HyAc*, and resulting in a *spontaneous increase in food intake*.

On admittance to a clinic, ANs would be randomly assigned to an experimental (EXP) or control (C) group. EXP ANs would engage in bed rest for most of the day/night, using an electric blanket and keeping the blanket’s temperature as high as is comfortable; ambient room temperature should also be elevated. In addition, they should be allowed to sit (with the heated blanket) and move around the clinic to a limited extent (partly to assess spontaneous movement and reduce musculoskeletal deterioration that occurs with bed rest). After baseline measurements are assessed on day 1, the following dependent variables should be *continuously* monitored in the EXP:

- T_core_ using ingested capsules (Bongers et al., 2018).
- Wrist skin temperature using a surface thermistor (Jacquot et al., 2014).
- A multi-sensor monitor (Sensewear Mini Armband) to monitor sleep patterns (Martinez-Sanchez et al., 2020; Hebebrand et al., 2023).
- A pedometer to monitor the amount of spontaneous walking.
- A heart rate monitor to ensure that ANs do not surreptitiously engage in moderate/high-intensity exercise.
- A modified shoe-based accelerometer to monitor foot and hand fidgeting (Belak et al., 2017).
- Three meals a day and snacks should be provided, and amounts eaten be recorded for spontaneous ingestion.
- An indwelling catheter to monitor daily changes in circulating hormones such as leptin and ghrelin, as well as reproductive and metabolic hormones.
- Assessment of body weight.
- Use of appropriate psychological questionnaires.

The C would be exposed to a treatment regime *typical for that particular clinic*. In addition, daily, T_core-low_, wrist skin temperature, total amount of movement, and food intake should be recorded.

The statistical analyses could involve a one-way ANOVA of the EXP group as well as a correlational analysis and multiple regression using all dependent variables. A 2-way ANOVA could be used to compare the results of the EXP and C groups.

In summary, it is proposed that the primary purpose of HyAc is to maintain T_core-low_. In addition, other positive benefits, such as reduced appetite and increased anxiolysis, also accrue; this assists in reinforcing HyAc. However, when viewed in terms of ancient times, HyAc also represents foraging/migration behavior, increasing the ANs’ chance of locating food (Guisinger, 2003). Regarding the effectiveness of heat as a treatment for ANs, definitive research is needed to determine whether heat application 24/7 in human ANs, initially reported by Gutierrez and Vazquez (2001) would produce a similar outcome as is seen in the ABA model (Gutierrez et al., 2002; Birmingham et al., 2004; Hillebrand et al., 2005).

### 5.2. Mood/attitude and psychological comorbidities during the acute phase of AN

The *buoyant mood and feelings of well-being* of the ANs correspond to her hypermobility, although, such behavior appears disproportionate to such severe weight loss (Casper and Davis, 1977; Casper, 2006, 2016, 2018). ANs seem to lack awareness of fatigue and weakness in the acute phase and insist on *not* being tired (Crisp, 1967; Casper, 2006; Hebebrand et al., 2023). Most maintain they never felt better (Casper, 1998). Until weight loss is profound, they deny feeling ill or fatigued (Vigersky et al., 1976) and are contented with their physical state (Casper and Heller, 1991). They also lack concern regarding the seriousness of the health risks of extreme weight loss (Casper and Heller, 1991; Gull, 1997). They continue to function by attending school and work, and many become enthusiastically involved in numerous activities (Casper, 2018).

Many researchers/clinicians have ascribed psychological comorbidities, such as depression, as a precipitating factor in AN (Blinder et al., 2006). However, Crisp (Crisp, 1984b) suggests that the degree of depression in AN is about the same as that found in other populations and is well below that in depressive illness. This is supported by Delvenne et al. (1992) who concluded that there is no association between AN and depression.

Others have concluded that there is no neurosis specific to anorexia nervosa (Kay and Leigh, 1954) and *no consistent precipitating* psychological pathology before the onset of AN (Bergh et al., 2013). Furthermore, most ANs initially seek medical attention because of drastic weight loss and not for psychological support.

If any psychological symptoms manifest, these are *preceded* by weight loss (Scolnick, 2018). Sodersten et al. (2016b, 2019) suggest that any *manifestation of psychological factors is related to the experience of semi-starvation* since this also occurs in healthy individuals exposed to extreme food restrictions. “It is possible that there are some ANs who have co-morbid depression, anxiety, obsessions, compulsions, or delusions. However, none of the 100 s of ANs we have treated to recovery have had such co-morbid disorders – their mental symptoms resolve as their eating behavior normalizes” (Bergh et al., 2013). After full clinical recovery, most reach complete psychological recovery (Vigersky et al., 1977; Bergh et al., 2002; Bulik et al., 2007; Sodersten et al., 2016b).

In summary, if present, many co-morbid symptoms are predominantly a result of the starvation response; “there is no need to postulate that mental factors predispose individuals to the illness” (Ioakimidis et al., 2011). However, it is acknowledged that the question of co-morbidities associated with AN remains controversial (Misra and Klibanski, 2014; Barakat et al., 2023).

### 5.3. ANs and disrupted sleep

In keeping with their energetic behavior and animated mood, ANs are restless sleepers (Hebebrand et al., 2023). Sleep disturbances are part of their high arousal pattern (Crisp et al., 1971; Kleppe et al., 2023). They have trouble falling asleep (Crisp et al., 1971); they wake more frequently during the night (Crisp, 1967; Crisp et al., 1971); these awake periods are more prolonged than those of HC (Crisp et al., 1971; Lacey et al., 1976). They also have a lesser total amount of sleep (Crisp et al., 1971; Lacey et al., 1976; Delvenne et al., 1992; Malcolm et al., 2022) and have significantly less REM sleep (Crisp et al., 1971). Another striking feature is that they wake early (Crisp et al., 1971). This insomnia is associated with low body weight; weight gain significantly improves many aspects of sleep (Lacey et al., 1976). Unlike severely depressed patients, ANs *rarely complain* of sleep problems (Crisp, 1980).

In a narrative review, Hebebrand et al. (2023) supported the findings that the majority of individuals with eating disorders suffer from sleep disturbances. This was more pronounced with binging/purging type ANs than restrictive AN. In addition, the *severity* of the eating disorder and activity levels were associated with sleeping disorders. They further reported that administration of human recombinant leptin, metreleptin, to five ANs, substantially improved sleep quality within one to 2 days; however, more research is needed to verify its usefulness.

Interestingly, sleep is closely aligned with thermoregulation in *healthy* adults (Jequier, 2002; Parmeggiani, 2003; Szymusiak, 2018). Krauchi (2007) has demonstrated that HCs (mainly women) who have trouble falling asleep, frequently have cold hands; he refers to this as “vasospastic syndrome.” This may account for the ANs delay in falling asleep, as she, too, has cold hands (Chudecka and Lubkowska, 2016).

It is also suggested that frequent wakings, longer times remaining awake, and early morning arousal may also be related to thermoregulation and sensations of coldness (Krauchi, 2007; Sauchelli et al., 2016), as well as unrelenting hunger (Goldstein et al., 2018). Cold sensations would encourage movement to increase metabolic heat and thus bolster the all-important T_core-low_, while hunger sensations would further motivate the need to forage for food.

In summary, ANs are HyAc, with the primary survival focus being maintaining T_core-low_. Their mood is upbeat and reflects their energetic behavior; this is associated with a disrupted sleep pattern compared to HCs.

## 6. SSs and “selection” of thermoregulatory behavior, coordinates with mood, and sleep

### 6.1. SSs and shallow torpor

SSs from the MinnSS, like ANs, have a T_core-low_ and experience extreme sensations of coldness. Like ANs, they need to reduce the discomfort of coldness and, in so doing, defend body temperature in the face of food deprivation. So, what behavioral thermoregulatory strategies do they adopt to satisfy these needs?

It is proposed that the primary behavioral thermoregulatory strategy “selected” by SSs is diametrically opposite to HyAc (Casper and Davis, 1977; Casper, 2022). SSs become tired, listless, and apathetic; these behaviors aim at conserving energy (Taylor and KEYS, 1950; Casper and Davis, 1977; Gorsky and Calloway, 1983; Hebebrand et al., 2022).

MinnSSs decreased the cost of daily activity from 1,567 to 451 Kcals per day, a 58% decrease (Franklin et al., 1948). During the first week of food reduction, SSs appeared fine; thereafter, overt movements became noticeably slower. “They climbed steps warily, one step at a time” (Taylor and Keys, 1950). One subject got stuck in the revolving doors of a downtown store and “never had the strength to push it open” (Taylor and Keys, 1950). In the later part of the 6-month fast, housekeeping chores and laboratory duties were neglected (Franklin et al., 1948). Participation in education programs for relief workers finally collapsed (Keys et al., 1950).

However, because of the requirements of the experimental condition, an overall level of activity *had to be maintained*. Subjects were obliged to walk to and from the mess hall, hike about 20 miles per week, and perform various other duties to simulate conditions of natural famine (Keys et al., 1950).

Although MinnSSs reported that physical exertion tired them and was avoided, occasionally, some exercised deliberately to lose weight to obtain extra bread rations or to prevent a reduction in food allocations because they had not lost enough weight (Keys et al., 1950). Some researchers have suggested that this additional activity implied that they remained “fairly active” (Epling and Pierce, 1996). On the contrary, all observations indicated that voluntary physical activity was markedly curtailed (Keys et al., 1950).

Similarly, decreased energy expenditure is a typical response to energy restriction in internment camps and during famines*: “*The first indications of a deficiency of food are languor, exhaustion, and general debility” (Franklin et al., 1948) “One can conclude that the natural tendency is to conserve energy during starvation by cutting down on all but a minimal level of essential activity” (Prentice et al., 1992). This decrease in energy expenditure reduces the depletion of stored fat reserves (Devlin, 2011).

Since SSs also need to maintain T_core-low_ but do not generate internal metabolic heat through physical activity, as do ANs, how do SSs maintain T_core-low_? It is proposed that the primary thermoregulatory behavior adopted by most undernourished, emaciated humans focuses on *retaining metabolic heat or passively gaining metabolic heat* instead of actively generating internal heat.

SSs wear additional layers of clothing to retain metabolic heat, use blankets, wraps, and the like, and assume specific body postures that reduce environmental exposure, such as curling up (Hart et al., 1962). They also use shelters (houses, huts, igloos) to protect themselves from the elements (Wyndham and Morrison, 1958). To *passively gain* metabolic heat, SSs absorb heat from external sources such as basking in the sun, taking long hot showers, using hot-water bottles and heaters, turning up the thermostat, making a fire, and eating *hot* food and drinks (Tucker, 2006). The *advantage* of this *inactive strategy* is that less stored energy (fat) is used.

Numerous mammals and birds conserve energy by hibernating during food shortages (frequently compounded by low ambient temperatures). Hibernation involves drastically reducing T_core_, BMR, and movement. This behavior may last weeks to months, reducing the energy required to sustain life. “Daily torpor” or “shallow torpor” is a less intense form of energy conservation compared to hibernation (Schmidt, 2014). *Shallow torpor* requires periods of inactivity throughout the day. It has *been defined as a temporary physiological state characterized by a controlled lowering of metabolic rate, body temperature, and physical activity below what is considered normal* (Melvin and Andrews, 2009). It is an evolved behavioral strategy to facilitate survival in response to food shortages (Berger, 1984) and is “the most effective means for energy conservation available to endotherms” (Geiser, 2013).

It is now proposed that SSs and other semi-starved victims engage in shallow torpor when confronted with food shortages and weight loss, frequently compounded by low ambient temperatures. Shallow torpor serves two functions: firstly, it reduces energy needs and thus *draws less* on stored body fat reserves. Secondly, it enables individuals to engage in passive heat-retention/acquisition behaviors.

### 6.2. SSs mood/psychological state

The mood of the MinnSSs corresponded with their reduced activity levels; they became depressed and apathetic, although individual differences were marked (Keys et al., 1950). Emotional and personality changes started developing within 2 months of the semi-starvation period. The humor and high spirits, which had been an outstanding quality of the group during the initial three-month Control Phase, gradually disappeared. They became irritable; some had temper outbursts and would sulk; a few had violent urges; they lacked self-discipline and self-control. They were indecisive, sensitive to noise, and unable to concentrate. They began spending more time alone, saying it became “too tiring” to contend with others. Some symptoms, such as moodiness and depression, became more severe as semi-starvation progressed. This occurred in subjects who were initially emotionally well-balanced and had “made as good an adjustment to life as people usually do.” (Keys et al., 1950).

Furthermore, Franklin et al. (1948) reported that certain subjects developed symptoms of “*semi-starvation neurosis*,” ranging in intensity from mild to severe. However, in most cases, these symptoms receded during the following rehabilitation period. Eventually, they returned to pre-starvation “normal,” although four of the 36 subjects were disqualified due to the development of semi-starvation-induced “*experimental neurosis*” (Kalm and Semba, 2005). This is supported by (Fessler, 2003).

### 6.3. SSs and sleep

The sleeping behavior of SSs also corresponded to their lethargic movements and mood. Although the length of sleep during the night was not changed in the MinnSS, “the men frequently took naps during the day” (Keys et al., 1950).

Keys et al. (1950) reported that in Prisoner of War Camps in WWII, the desire for sleep increased; the number of hours that an adult male would wish to remain in bed, partly dozing but mostly in genuine deep sleep, steadily rose from 8 h to 16 h or more out of the 24 h. During sleep, BMR and body temperature decrease, and there is reduced movement; these adaptations further decrease energy needs (Schmidt, 2014; Monnard et al., 2017).

The following example illustrates how a group of present-day Russian peasants has learned to deal with food shortages and cold ambient temperatures; this custom has existed since time immemorial (Human Hibernation, 2000): “At the first fall of snow, the whole family gathers around the stove, lies down, ceases to wrestle with the problems of human existence, and quietly goes to sleep. Once a day, everyone wakes up to eat a piece of hard bread, of which an amount sufficient to last 6 months has providently been baked in the previous autumn. When the bread has been washed down with the draught of water, everyone goes to sleep again. The members of the family take … turn [s] to watch and keep the fire alight. After 6 months of this reposeful existence, the family wakes up, shakes itself, goes out to see if the grass is growing, and by-and-by sets to work at summer tasks.”

**Figure 1:**
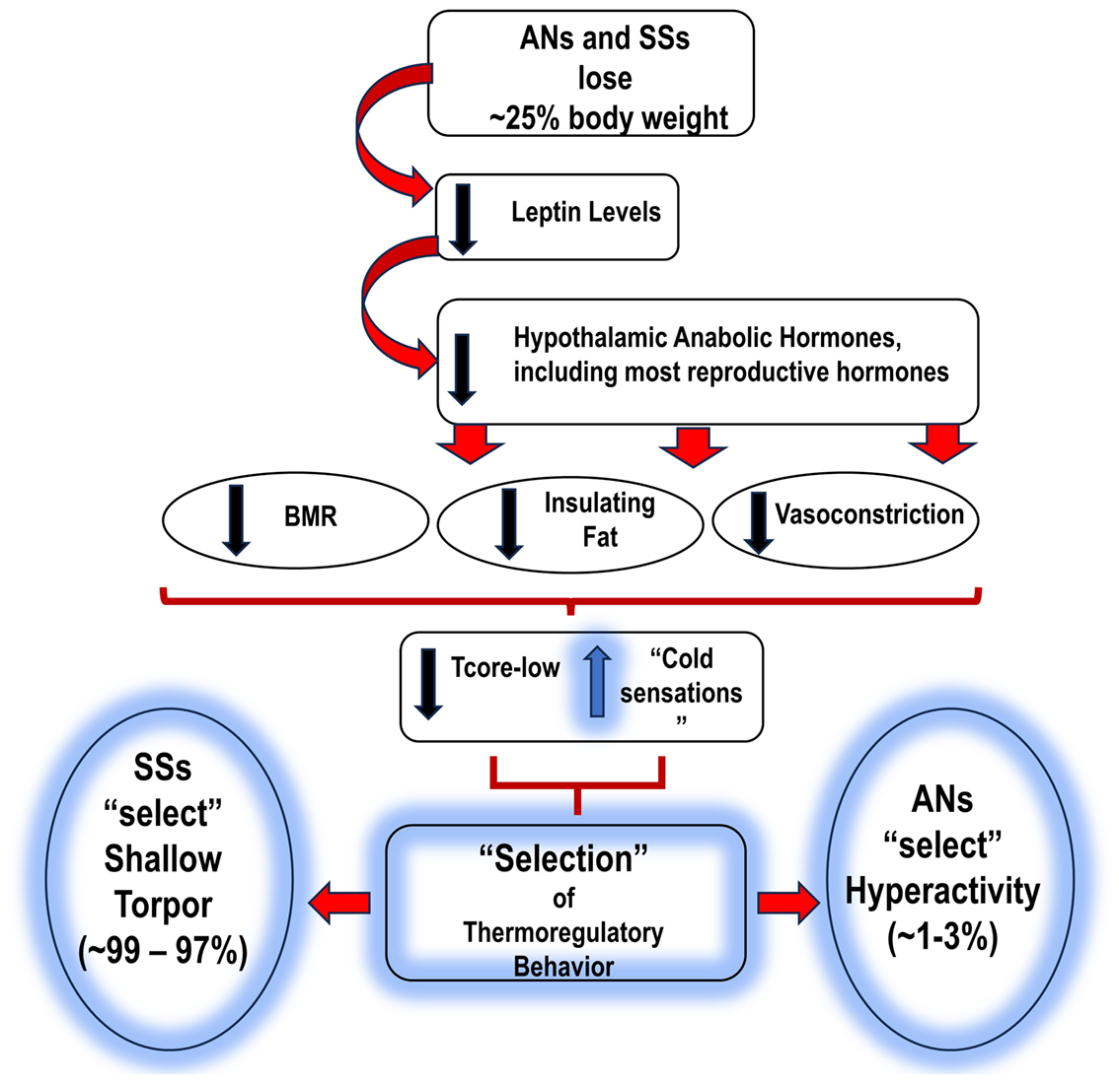
Proposed sequence of events leading to AN and SS, and the subsequent “selection” of thermo-behaviors. AN, anorexia nervosa; SS, semi-starvation; BMR, basal metabolic rate; T_core-low_, reduced body temperature; HyAc, hyperactivity.
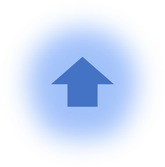
 = increase. 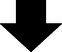 = decrease.

In summary: to deal with the two issues of feeling cold/maintenance of T_core/low_ and reduced food availability, SSs focus on being inactive; this behavioral pattern is referred to as shallow torpor. In keeping with this strategy, SSs demonstrate a depressive mood and extended hours of sleep (Figure 1).

## 7. Vignette

The following hypothetical scenario is presented to contextualize the above ideas.

From about 2 million years ago to about 12,000 years ago, hunter-gatherer culture was humans’ only way of life (Fessler, 2002; Guisinger, 2003). ANs and SSs thermo-behavioral “strategies” were most likely operational during these times (Fessler, 2002; Guisinger, 2003).

To illustrate responses to reduced food availability and cold temperatures, suppose, ~14,000 years ago, a Clan of hunter-gatherers lived in a temperate region of the Northern Hemisphere with hot summers and cold winters (Guisinger, 2003; Vazquez et al., 2006b; Scolnick and Mostofsky, 2015). This Clan possibly consisted of ~70 individuals, including 30 females.

To obtain food, males hunted larger game, and women and children foraged for berries, fruits, bulbs, and the like (Fessler, 2002). As a result, everyone was well-fed in summer and most likely built-up excess body fat in preparation for winter shortages.

As winter approached and ambient temperatures declined, hunted, and foraged food became scarce. The men ventured out less frequently as game became sparser; the group became more reliant on the 30 women, the primary foragers (Fessler, 2002). Since foraged food was gradually depleted in nearby areas, the women were obliged to walk *greater distances* but collected *less food*.

Foraging was an energy-intensive activity (Pontzer et al., 2012). Thus, all 30 females began losing weight over subsequent weeks and months due to increased energy expenditure and reduced caloric consumption. Eventually, all 30 females lost approximately 25% of body weight; associated with this weight loss was a reduction in metabolic rate, T_core_, and related changes (Hebebrand et al., 2022). All 30 females now faced two primary survival challenges: *reduced availability of food and maintenance of T_core-low_,* probably exacerbated by decreases in ambient temperatures (McCue, 2010).

With this weight loss, it is proposed that foraging became unsustainable *for 29/30* women (~99%). Most became lethargic and adopted the strategy of shallow torpor. They winterized shelters, remained in caves, used animal skins to keep warm, built fires, and slept for extended periods. Most of their energy was derived from stored body fat.

The 30^th^ woman (1%), with a genetic predisposition to AN, adopted a diametrically opposite strategy: she became hyperactive. This hyperactivity would help maintain her T_core-low_ and increase her chances of locating food. The problem with this restless behavior was that it was highly energy-consuming, necessitating increased foraging and, in some cases, migrating to warmer regions where more food was available (Guisinger, 2003). AN migration most likely *contributes* to the fact that female DNA is more diverse in many groups than male DNA (Seielstad et al., 1998).

With the onset of warmer weather and food availability, SSs and ANs would *regain lost weight.* In ANs, this would reduce HyAc, normalize T_core-low_, and re-establish mood and sleep patterns. In SSs, shallow torpor would diminish, and they, too, would re-establish “normal” behaviors. When weight was regained, possibly more fat would accumulate in both groups (Dulloo et al., 1996; El Ghoch et al., 2014); this would better prepare these females for a possible repeat scenario. From this perspective, changes in ANs and SSs may be regarded as *seasonal* (Gutierrez et al., 2013; Scolnick and Mostofsky, 2014, 2015).

In modern-day ANs, despite the frequent abundance of food and availability of heat, ANs *inappropriately express HyAc behavior*. It is proposed that this behavior remains a *vestigial substitute for temperature regulation and foraging.* In contrast, SSs do not adopt a strategy of shallow torpor in the presence of warmth and copious food.

## 8. Conclusion

In answer to Phillipou et al.’s (2018) question of whether psychologists are treating AN or SS, it is proposed that the *initial* physiological response to reduced caloric intake and weight loss in both groups are *similar*. The organism does not distinguish between weight loss due to willful food refusal or externally imposed circumstances such as famine. These physiological alterations aim at extending survival (Devlin, 2011).

It is proposed that these changes represent homeorhesis (as opposed to homeostasis). Homeorhesis reflects *an orchestrated change during a specific physiological state,* such as during a 9-month pregnancy (Bauman and Currie, 1980) or in association with injury and/or infection and the manifestation of “sickness behavior” (Shattuck and Muehlenbein, 2015). “*The body adopts alternate strategies* to *promote optimal functioning during atypical circumstances.*” (Bauman and Currie, 1980). When the cause for these changes is eliminated (in the case of ANs and SSs this would be increased caloric consumption and weight gain), the body reverts to its “standard” homeostatic state (Bauman and Currie, 1980).

**Figure 2:**
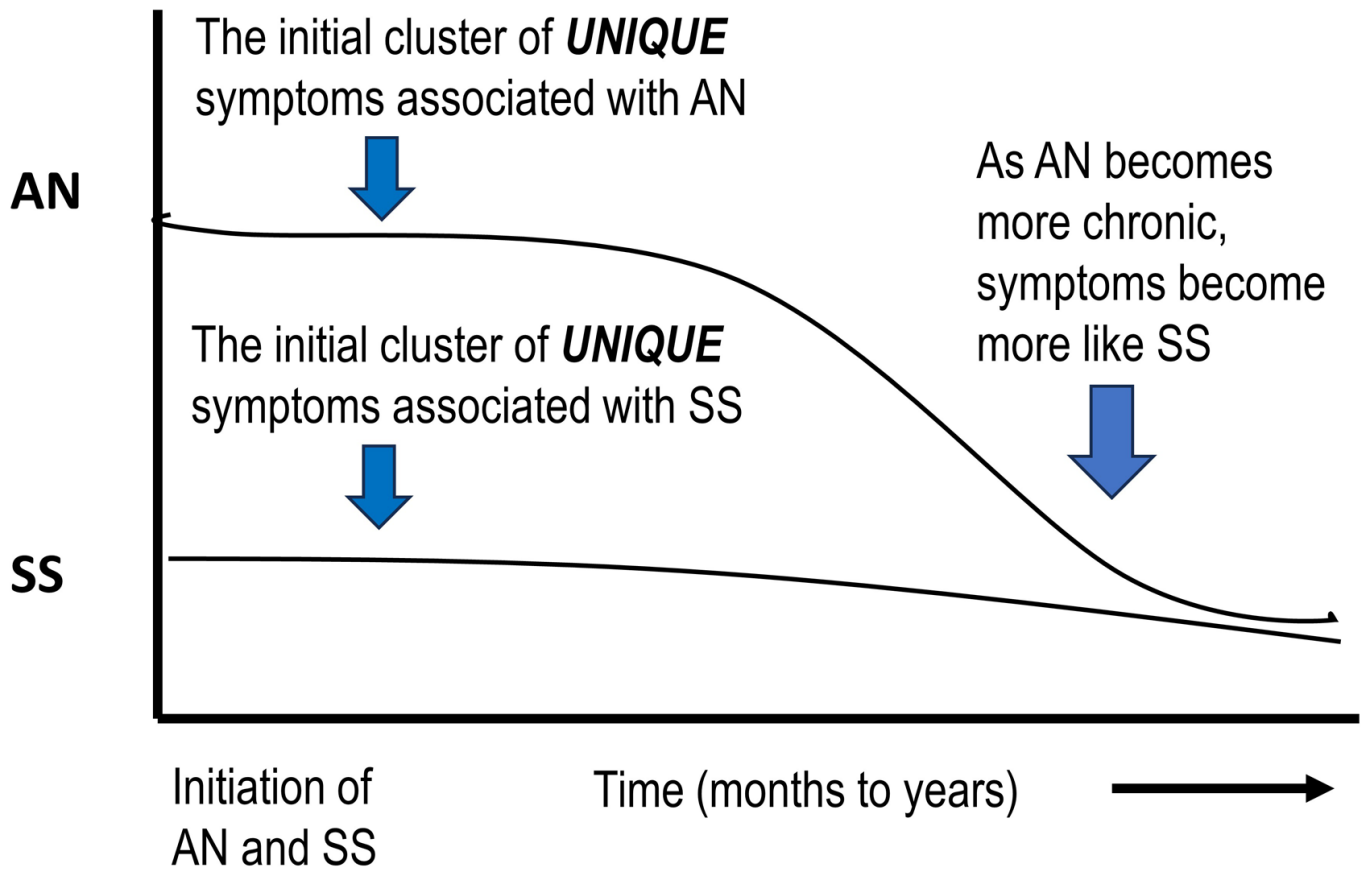
Schematic diagram suggesting that if acute AN becomes chronic, symptoms will more closely resemble SS. AN, anorexia nervosa; SS, semi-starvation.

The *primary differences* between ANs and SSs represent different strategies that each adopts to promote the maintenance of body temperature. For ANs this includes hyperactive behavior, an energetic mood, and reduced sleep. For SSs, behavior becomes lethargic; they display a depressive mood and an extensive increase in sleep. These thermo-behavioral “selections” are most likely genetically based, with the genetic component in ANs ranging from 32 to 76% (Bulik et al., 2019; Steiger and Booij, 2020; Barakat et al., 2023). Such changes could involve central re-programming of brain neurotransmitters, neuromodulators, and associated receptors (Pirke et al., 1984; Fessler, 2002; Levine and Kotz, 2005; Teske et al., 2008; Sodersten et al., 2016b; Zup et al., 2022; Barakat et al., 2023). Although there is considerable information on central changes in ANs, there is less information on these factors in SSs, possibly since their response to semi-starvation seems to be a “natural” adjustment (Figure 2).

Although behavioral differences are proposed during *acute* AN, it is suggested that if this condition continues for an extended period, *acute AN will progress into a chronic state* (Crisp, 1984b; De Filippo et al., 2016). At this point, psychologists are probably treating SS and associated symptoms as well as inappropriate habit formation (Uniacke et al., 2018). If this assumption is correct, this would *stress the importance of early detection and intervention* (Kaye and Bulik, 2021; Koreshe et al., 2023).

Finally, it is suggested that the “adoption” of shallow torpor by SSs may be a more successful survival strategy than ANs’ hyperactive strategy. The rationale for attesting to this is that the number of females who “practiced” shallow torpor during times of food shortages and wintry weather *appears* to be much larger (96–99.09%) than ANs who adopted the strategy of HyAc (0.91–4%; Watson et al., 2019).

## Data availability statement

The original contributions presented in the study are included in the article/supplementary material, further inquiries can be directed to the corresponding author.

## Author contributions

The author confirms being the sole contributor of this work and has approved it for publication.

## Abbreviations

MinnSS(s): Minnesota Study of Semi-Starvation (*subjects*)
AN(s): anorexia nervosa (*patients*)
SS(s): semi-starved (*individuals*)
HCs: healthy controls
HyAc: hyperactivity
